# Social trust and COVID-19 mortality in the United States: lessons in planning for future pandemics using data from the general social survey

**DOI:** 10.1186/s12889-024-19805-y

**Published:** 2024-08-27

**Authors:** Megan E. Marziali, Robert S. Hogg, Alexi Hu, Kiffer G. Card

**Affiliations:** 1 Department of Epidemiology, Mailman School of Public Health, Columbia University, New York, USA; 2 Faculty of Health Sciences, Simon Fraser University, British Columbia, Canada; 3 British Columbia Centre for Excellence in HIV/AIDS, British Columbia, Canada

**Keywords:** COVID-19, Social trust, Collectivism, Social disconnectedness, Loneliness

## Abstract

**Background:**

The United States has lost many lives to COVID-19. The role of social capital and collective action has been previously explored in the context of COVID-19. The current study specifically investigates the role of social trust at the county level and COVID-19 mortality in the US, hypothesizing that counties with higher social trust will have lower COVID-19 mortality rates.

**Methods:**

We used cross-sectional data from the General Social Survey (GSS). We collected COVID-19 mortality data from the COVID-19 Dashboard by the Center for Systems Science and Engineering (CSSE) at Johns Hopkins University until October 31, 2021. We obtained county characteristics from the 2019 American Community Survey and supplemented this data source with additional publicly available county-level data, such as measures of income inequality and political leanings. We measured social trust as a single item from the GSS and calculated mean social trust in a county by pooling responses from 2002 to 2018. We then modeled the relationship between mean social trust and COVID-19 mortality.

**Results:**

Results indicate that counties with higher social trust have lower COVID-19 mortality rates. Higher values of mean social trust at the county level are associated with a decrease in COVID-19 mortality (b= -0.25, *p*-value < 0.001), after adjustment for confounding. The direction of association is consistent in a sensitivity analysis.

**Conclusions:**

Our findings underscore the importance of investment in social capital and social trust. We believe these findings can be applied beyond the COVID-19 pandemic, as they demonstrate the potential for social trust as a method for emergency preparedness.

**Supplementary Information:**

The online version contains supplementary material available at 10.1186/s12889-024-19805-y.

## Introduction

The COVID-19 pandemic claimed many lives in the United States, both in terms of deaths directly attributable to COVID-19 and excess deaths indirectly attributable to COVID-19 [1, 2]. While scientific advancements, such as the development of vaccines and rapid testing, are conducive to substantially reducing COVID-19 mortality rates, psychosocial factors also play a role in pandemic preparedness and infectious disease burden. The COVID-19 pandemic highlighted the importance of collectivist actions for disease prevention. Public health measures to prevent COVID-19 infections relied on group collectivism, consisting of both group- and individual-level precautions. Group-level measures acting at the policy level can consist of mechanisms such as enforcing a mask mandate, stay-at-home guidelines, and requiring a COVID-19 test or proof of vaccination before travel. Individual-level actions to protect themselves and others from COVID-19 include, but are not limited to, methods such as: getting vaccinated against COVID-19, wearing a mask in high-risk spaces, physical distancing, and testing for COVID-19 when sick.

Researchers explored the role of various dimensions of social capital on COVID-19 mortality, with some inconsistent findings. For example, time-series analyses in 84 countries, including countries in world regions such as the Middle East, Africa, North and South America, and others, suggest that countries lacking in some measures of social capital experienced more COVID-19 deaths [3]. Similarly, research conducted in the US suggests that higher levels of social capital are protective against COVID-19 infection and mortality [4]. However, additional work demonstrated that specific dimensions of social capital were either positively (community attachment and social trust) or negatively (family bond and security) associated with COVID-19 deaths [5]. 

These studies point towards a need for investigating and focusing on specific dimensions of social capital. Our study specifically considers social trust, a component of social capital facilitating resource-sharing [6, 7], essential for collectivist action within a community or network group [6]. Whether a society has high levels of interpersonal trust is related to how it can engage in collective action [8, 9] and can increase willingness to support public health measures and services [10–12]. This is exemplified by work conducted in a Canadian setting that emphasizes the importance of collectivist action by indicating that collectivism is associated with abiding by stay-at-home ordinances [13]. 

The role of interpersonal trust, specifically in the context of the COVID-19 pandemic, has been explored globally. Some findings suggest that higher interpersonal social trust is associated with higher COVID-19 mortality rates, through a pathway involving more social interactions [3, 5]. This is in contrast to a global study finding that higher interpersonal trust and government trust are associated with lower COVID-19 infection rates [14]. Data from Sweden suggest that non-compliance with COVID-19 public health measures is associated with lower social trust; [15] this is in line with studies in the global context, which find higher interpersonal trust to be associated with increased compliance with COVID-19 public health measures, such as vaccine coverage [14, 16, 17]. A cross-national analysis found that, while a higher level of social trust may result in a shorter duration to the first outbreak due to more cohesive social relationships and lower risk perception, social trust may facilitate cooperation and compliance to neutralize COVID-19 [18]. 

The complex interplay of interpersonal and institutional trust further complicates the relationship between social trust and COVID-19 deaths. The role of institutional and interpersonal trust are both underscored as drivers of COVID-19, with interpersonal trust invoked as a leverage point for participation in mitigation policies such as decreased outdoor activity [19]. Scholars further highlighted the importance of political trust by examining the role of partisanship in compliance with COVID-19 mitigation policies, finding that Republican-leaning areas complied less than those leaning Democrat; [17] they further suggest that social capital is protective in mitigating COVID-19 cases in liberal counties but a risk factor in conservative areas [20]. Researchers described the interaction of political and interpersonal trust in complying with social distancing measures, noting inconsistent findings over time [21]. 

There is precedence to believe that social trust influences outcomes during the COVID-19 pandemic, potentially through collectivist action. In light of conflicting findings, this study explores the role of social trust in COVID-19 deaths at the state level in the United States. We hypothesize that areas with a higher social trust will have lower COVID-19 mortality rates.

## Methods

### Participants and recruitment

Data are from the National Opinion Research Council (NORC) General Social Survey (GSS), a nationally representative panel survey within the United States. Using multi-stage area probability sampling, participants are recruited for the GSS every two years (with some exceptions) from 1972 to 2018 [22]. Detailed information concerning sampling methods is available elsewhere [22]. This cross-sectional analysis pooled data from the 2002–2018 surveys, capturing a total of 19,712 participants. We conducted sensitivity analyses restricted to pooling data from the 2016–2018 GSS to determine whether this impacted variability in our effect estimate.

All statistical analyses were conducted in R using R-Studio. The data were filtered to include counties with at least more than one observation.

### Exposure: social trust

We defined social trust through a survey question asking participants whether *“Generally speaking*,* would you say that most people can be trusted or that you can’t be too careful in dealing with people?”*. Response categories included ‘most people can be trusted,’ ‘can’t be too careful,’ or ‘it depends’ [23]. We calculated the intraclass coefficient (ICC) to explore clustering at the county level in the GSS data. We found an ICC of 0.22, indicating clustering was a concern; as such, we averaged the measure of social trust to the county level, allowing social trust to be interpreted as the mean level of social trust per county. The mean social trust in a county was calculated by assigning levels of social trust the following numeric values: 0 = ‘can’t be too careful,’ 0.5 = ‘it depends,’ 1.0 = ‘most people can be trusted.’ We pooled data from 2002 to 2018 to estimate mean social trust in a county; we conducted sensitivity analyses restricting to 2016–2018, to test the stability of the measure of mean social trust and its effect on COVID-19 mortality.

### Outcome: COVID-19 mortality

We used data from the COVID-19 Dashboard by the Center for Systems Science and Engineering (CSSE) at Johns Hopkins University to amass information on COVID-19 cases and deaths [24]. COVID-19 deaths at the county level were counted up until October 31, 2021. The death rate per 1000 individuals was calculated by dividing the number of deaths in a county by the total population of the county and multiplying this value by 1000.

### Covariates

Using information from the 2019 American Community Survey (ACS) [25], we adjusted for county characteristics, including average household size and population density. We also adjusted for county-level sociodemographic characteristics, including median age, percentage of the population that is white, median income, and percentage of the population that is insured. We adjusted for income inequality using an index for income inequality available from the 2020 County Health Rankings & Roadmaps (CHRR) [26] and the proportion of Republican voters, from the 2020 election results [27]. All data were harmonized at the county level through the Federal Information Processing Standards (FIPS) codes [28]. 

### Statistical analysis

We first performed bivariate analyses to investigate the relationship of each covariate with the outcome, COVID-19 mortality. To test the relationship between these mean social trust in a county and the county’s death rate, we fit a Generalized Linear Model (GLM) with a Gaussian family using the ‘glm’ function, controlling for median income (per $10,000 increase), median age (per 10-year increase), percentage insurance, percentage of residents who were white, percentage of residents that voted republican, population density of the county per square mile (per 100 people), and inequality. The dependent variable in the model was the death rate and the primary explanatory variable was mean social trust. The model was weighted using survey weights [29]. All models were adjusted for a priori hypothesized confounders of the relationship between social trust and COVID-19 deaths. We removed participants with missing data or who lived in counties with missing data from our analyses. Due to missingness, 10,963 observations were analyzed from 2002 to 2018, with participants representing 374 counties.

## Results

### Descriptive statistics

Table 1 contains descriptive statistics for participants from the pooled 2002–2018 General Social Survey. The analytic sample included 10,963 participant responses. Of these participants, 61.8% reported distrusting others, with 33.0% reporting that they trust others. Respondents were predominantly female (55.4%), White (76.4%), and currently married (45.7%). Most participants were either in the middle class (42.4%) or working class (45.7%).

**Table 1:** Sample characteristics of participants included in the 2002–2018 General Social Survey (*N* = 10,963)

Variable	Overall (*N* = 10,963) %
Social trust	
Distrust	61.8
Trust	33.0
Depends	5.2
Sex	
Male	44.6
Female	55.4
Subjective social class	
Lower class	8.8
Middle class	42.4
Working class	45.7
Upper class	3.0
Marital status	
Currently married	45.7
Divorced	16.5
Never married	25.9
Separated	3.4
Widowed	8.6
Race	
Black	15.2
White	76.4
Other	8.5

Table 2 contains descriptive statistics for county-level characteristics. On average, counties had a median age of 39, were 74% white, with 48% of the county voting Republican.

**Table 2:** County-level characteristics for counties with participants who responded in the 2002–2018 general social survey

Variable	Overall Mean (S.E.)
Mean social trust	0.37 (0.27)
Median income per 10,000USD	8.24 (2.13)
Median age	39.01 (4.10)
Proportion of population: Insured	0.90 (0.05)
Proportion of population: White	0.74 (0.17)
Proportion of voters: Republican	0.48 (0.17)
Population density (per mile^2^)	17.46 (48.16)
Income inequality index	4.67 (0.84)

S.E.: Standard Error

### Multivariable models

After adjustment for appropriate county-level confounders, our results indicate that counties with higher levels of social trust, as measured in the pooled 2002–2018 data, had lower COVID-19 mortality (Table 3). We report our sensitivity analysis, restricted to 2016–2018 data for calculating mean social trust, in Table S1. Broadly, analyses show that this restriction in years of data in calculating mean social trust did not change our conclusions regarding the association between social trust and COVID-19 mortality; however, the effect estimate is stronger (Table S1).

**Table 3:** Generalized linear model estimating the relationship between COVID-19 deaths per 1,000 population at the county level with mean social trust measured from 2002–2018

Variable	Estimate	Standard Error
***Main effect***		
Mean social trust	-0.25***	0.07
***County-level confounders***		
Median income (per 10,000 USD)	-0.03**	0.01
Median age	0.04***	0.004
Proportion of population: Insured	-0.47	0.48
Proportion of population: White	-2.25***	0.19
Proportion of voters: Republican	3.27***	0.19
Population density (per mile^2^)	0.002***	0.00
Income inequality index	0.47***	0.03

*p-value < 0.05, ** *p*-value < 0.01, ***p-value < 0.001

## Discussion

This paper explores the role of social trust in COVID-19 mortality. Our results indicate that counties with higher levels of social trust have lower COVID-19 mortality rates. We believe that findings from this study highlight the importance of investment in building social capital and trust beyond the COVID-19 pandemic. Previous research details the importance of collectivism and social capital in mitigating the harmful effects of pandemics, such as Ebola [30, 31], and other natural disasters [32]. Further investigations should explore how investment in social trust could be used as a method of emergency preparedness for future pandemics and widespread crises.

Social capital is a protective factor in various health-related outcomes; [33] it follows that social trust, a component of social capital, is also associated with health-related outcomes across societal contexts. Prior work shows that social trust is associated with all-cause mortality within the U.S., hypothesized to operate biologically through stress and the hypothalamic-pituitary-adrenal (HPA) axis [23]. A small study (*N* = 20) conducted in Japan with males ages 19–23 found that people who reported higher levels of interpersonal trust had lower levels of cortisol, a chemical associated with stress responses [34]. A similar study in the same context found higher social trust, as a function of social capital highly related to income inequality, to be associated with lower mortality, including all-cause mortality, coronary heart disease, malignant neoplasms, and infant mortality [35]. Community-level social trust among adults in Korea is associated with a lower risk of death from all causes, cardiovascular disease, and cancers [36]. 

People who are socially disconnected, or, lonely, tend to have lower levels of social trust [37]. Loneliness is associated with increased risk of mortality [38, 39] and adverse health outcomes, including but not limited to psychiatric disorders [40] and cardiovascular diseases [41]. As briefly described above, the mechanism of action is hypothesized to operate through allostatic load and the HPA axis; [23] thus, people who are lonely tend to have higher levels of inflammation [42]. The relationship between loneliness, social trust, inflammation, and adverse health outcomes could suggest that people with a lower degree of social trust were more susceptible to COVID-19 disease. This is bolstered by findings suggesting that inflammation as a result of chronic stress acts synergistically with inflammation from COVID-19 to produce poor health outcomes [43]. Lastly, recent research suggests that lower social cohesion is associated with a lower antibody response from COVID-19 [44], emphasizing this link between social and physical health.

To better understand the importance of the association between social trust and mortality, we must first outline the landscape of social trust within the U.S. Levels of social trust were already decreasing in the U.S. prior to the COVID-19 pandemic. A survey conducted by the Pew Research Center found that 79% of U.S. adults believe they have little confidence in each other (a measure of interpersonal trust), with 58% reporting that it is important to improve levels of confidence between U.S. adults [45]. Research indicates that, historically, levels of social trust vary widely within the U.S.; a higher lack of social trust correlates with greater age-adjusted mortality rates at the state level [35, 46]. Income inequality, a relevant component when discussing income gradients in COVID-19 deaths, is also correlated with a lack of social trust, wherein greater income inequality is associated with lower levels of social trust [35]. 

The COVID-19 pandemic impacted two facets of social trust: neighborhood and generalized trust. A study with Canadian data conducted during the pandemic delineates distinct trajectories of social trust and found that trust either decreased, increased, or remained stable [47]. Socioeconomic position interacted with social trust to lead to discrepancies in trajectories, with increases in trust observed among people in a higher socioeconomic position and decreases among people in a lower socioeconomic position [47]. Complicating this relationship is data from Sweden, which indicates greater non-compliance in public health measures among people in higher socioeconomic positions [15]. The income gradient in COVID-19 mortality has been well established across various contexts, with higher mortality rates in lower income groups and among people with higher sociodemographic risk factors [48, 49]. Further, counties with greater income inequalities in the United States had higher COVID-19 mortality [50, 51]. While data originated from different countries, these findings suggest a potential mechanism wherein non-compliance with public health measures in conjunction with lower mortality, both in higher socioeconomic groups, may be driving lower social trust among people in lower socioeconomic positions. Future research should delineate the relationship between income inequality and social trust within the US.

This analysis has important limitations to consider. This is a cross-sectional analysis, and social trust was measured at a single point in time for each participant; as such, it is not possible to conclude whether COVID-19 increased or decreased social trust. It is feasible that levels of social trust varied between the time when responses were collected and the onset of the COVID-19 pandemic. Additionally, it is feasible that people who are socially connected versus disconnected contracted COVID-19 at different time points throughout the pandemic. A time series analysis is warranted in future studies, to further our understanding of trajectories of COVID-19 infection and mortality across the spectrum of social trust. While we included a priori hypothesized confounders, we cannot eliminate nor rule out unmeasured or residual confounding. Lastly, measures of social trust are likely not generalizable at the county level and are subject to change over time. Future work should investigate social trust in differing contexts and at multiple levels of social organization, given regional variations in social trust [52]. Exploring social trust across countries in different world regions is important for understanding the complexity of the relationship between social trust and COVID-19 outcomes; while there is a dearth in the literature concerning social trust in low- and middle-income countries [53], examining social trust and COVID-19 outcomes within low- and middle-income countries would also help to inform how investment in building social trust could prevent future pandemics.

## Conclusions

Using data from the pooled 2002–2018 General Social Survey, we find that higher mean social trust is associated with lower COVID-19 mortality. A complex interaction between non-compliance with public health measures in conjunction with income inequality could explain our results. We believe these findings highlight the importance of social trust as a means of emergency preparedness. Further exploration into the investment of social trust within communities as a prevention measure for future pandemics is warranted.

### Electronic supplementary material

Below is the link to the electronic supplementary material.

Supplementary Material 1

## Data Availability

This study uses secondary data publicly available from: the National Opinion Research Council General Social Survey (GSS) (https://gss.norc.org/Get-The-Data); the Center for Systems Science and Engineering (CSSE) at Johns Hopkins University (https://coronavirus.jhu.edu/map.html); the American Community Survey (https://www.census.gov/programs-surveys/acs); the County Health Rankings & Roadmaps (CHRR) (https://www.countyhealthrankings.org); and, the 2020 US presidential election results, available from GitHub (https://github.com/tonmcg/US_County_Level_Election_Results_08-20). The authors do not own nor control these datasets.
