# Supplementary material for: Social trust and COVID-19 mortality in the United States: lessons in planning for future pandemics using data from the general social survey
Source: BMC Public Health. 2024 Aug 27;24:2323. doi: 10.1186/s12889-024-19805-y (PMC11348587; doi:10.1186/s12889-024-19805-y)
Supplement: Supplementary file 1 — Supplementary Material 1 [file 12889_2024_19805_MOESM1_ESM.docx]

**SUPPLEMENTAL TABLES**

**Table S1.** Generalized linear model estimating the relationship between COVID-19 deaths per 1,000 population at the county level with mean social trust measured from 2016-2018.

| **Variable** | **Estimate** | **Standard Error** |
| --- | --- | --- |
| ***Main effect*** |  |  |
| Mean social trust | -0.42** | 0.15 |
| ***County-level confounders*** |  |  |
| Median income (per 10,000 USD) | -0.04 | 0.02 |
| Median age | 0.04*** | 0.01 |
| Proportion of population: Insured | -0.24 | 1.07 |
| Proportion of population: White | -2.33*** | 0.41 |
| Proportion of voters: Republican | 2.94*** | 0.43 |
| Population density (per 100 mile^2^) | 0.002* | 0.001 |
| Income inequality index | 0.45*** | 0.06 |

*p-value < 0.05, ** p-value < 0.01, ***p-value < 0.001
